# Temperature estimation of a pair of trapped ions

**DOI:** 10.1038/s41598-022-10572-7

**Published:** 2022-04-23

**Authors:** O. P. de Sá Neto, H. A. S. Costa, G. A. Prataviera, M. C. de Oliveira

**Affiliations:** 1 Coordenação de Ciência da Computação, Universidade Estadual do Piauí, Parnaíba, PI 64202–220 Brazil; 2 Departamento de Física, Universidade Federal do Piauí, Teresina, PI 64049-550 Brazil; 3 Departamento de Administração, FEA-RP, Universidade de São Paulo, Ribeirão Preto, SP 14040-905 Brazil; 4 Instituto de Física Gleb Wataghin, Universidade Estadual de Campinas, Campinas, SP 13083-970 Brazil

**Keywords:** Optics and photonics, Physics

## Abstract

We apply estimation theory to a system formed by two interacting trapped ions. By using the Fisher matrix formalism, we introduce a simple scheme for estimation of the temperature of the longitudinal vibrational modes of the ions. We use the ions interaction to effectively infer the temperature of the individual ions, by optimising the interaction time evolution and by measuring only over one of the ions. We also investigate the effect of a non-thermal reservoir over the inference approach. The non-classicality of one of the ions vibrational modes, introduced due to a squeezed thermal reservoir, does not directly affect the inference of the individual temperatures, although allowing the modes to be entangled. To check actual experimental conditions, we analyze the temperature inference under heating due to surface-electrode noise.

## Introduction

In the quantum mechanical context, the temperature of a system is a nonlinear function of the density operator, so it cannot directly correspond to a quantum observable—one has to indirectly estimate its value by measuring another observable. This indirect procedure for temperature estimation implies in an additional uncertainty for the measured value, which should be handled appropriately. Therefore, any strategy aimed to determine the temperature of a quantum system reduces to a parameter estimation problem^1–3^. Quantum theory of estimation (QET) provides a formal framework in order to optimise the inference procedure by minimising the additional uncertainty^4,5^.

Techniques of quantum parameter estimation have been devoted to estimate the temperature in the context of quantum thermodynamics^6–9^ and also for technological applications in many different branches of Science—ranging from Material Sciences to Biology and Medicine^10,11^. A central purpose is to employ a controlled quantum system (with view to applications in low temperature measurement^12^) to explore the thermodynamics in the regime of small-scale physics^13–16^, where quantum effects become predominant^17–19^. Several quantum systems have been used to estimate very low temperature such as Bose-Einstein condensates^20^, ultracold lattice gases^21^, trapped ions^22–24^, single-qubit^6^, to mention a few.

In this work, we address the issue of the vibrational degrees of freedom temperature estimation in a system of two interacting trapped ions. Trapped ion systems constitute an outstanding platform for observing fundamental physical phenomena and has surfaced as one of the central potential architectures for implementing scalable quantum computation^25,26^ and quantum simulation^27–29^. In those systems, the ions are trapped by oscillating electromagnetic fields in linear or surface-electrode Paul Traps and are held for manipulations at cryogenic temperatures. Usually, fluctuations in the electromagnetic fields introduce perturbations in the equilibrium state of each distinct ion, causing inhomogeneity in the ions temperatures and spurious heating. Therefore, to access the individual ions temperatures is fundamental for the establishment of optimal conditions for the ions manipulation. Our interest here is to investigate how precisely we are allowed to estimate the local temperatures of the vibrational degree of freedom of the two ions simultaneously. For this purpose, we employ the multiparameter Fisher information

1 $$\begin{aligned} {\mathscr {F}}_{\alpha \beta } = \sum _{r_i} {\mathscr {P}}(r_i)\left( \frac{\partial \ln {\mathscr {P}}(r_i)}{\partial \theta _\alpha }\right) \left( \frac{\partial \ln {\mathscr {P}}(r_i)}{\partial \theta _\beta }\right) , \end{aligned}$$

to investigate the parameter estimation accuracy^30–32^, where $\theta _i$ denotes the outcome of a measurement, ${\mathscr {P}}(r_i)=p(r_i|\theta _i)$ is the conditional probability of measuring $r_i$ if the value of the parameter under consideration is $\theta _i$. From the practice standpoint, increasing the Fisher information of the system tends to increase the maximum precision that can be obtained by an estimation scheme. Mathematically, this relationship is quantified by the Cramér-Rao lower bound^33,34^,

2 $$\begin{aligned} \mathrm {Var}({\hat{\theta }}_i) \ge \frac{1}{\sqrt{{\mathscr {F}}_{ii}}}, \end{aligned}$$

where ${\hat{\theta }}_i$ is the estimator of the unknown parameter $\theta _i$. The Cramér-Rao lower bound is saturated asymptotically by the optimization of the elements of the Fisher information matrix via a suitable choice of all its dependent parameters. Quantum parameter estimation purpose in quantum thermometry is to use a measurement over a quantum probe system, to infer the temperature of the reservoir it is immersed. For that purpose, an optimisation over the measurement operation is usually employed in order to find the maximal overall Fisher information (and consequently the measurement with the minimal dispersion). However, the procedures employed, are sometimes cumbersome and quite generally do not bring any information on how and which observable is to be measured^12^. Such a difficulty is even worse in the situation when systems of continuous variables are involved^16,35,36^. Here we take a more pragmatical approach, instead of optimising the measurement operator we employ measurements accessible in actual ion experiments^37,38^ for the calculation of the Fisher information. We employ the detection of one of the ions vibrational mode phonon number, which is accessed experimentally, through the observation of the asymmetry between the red and blue motional sidebands of hyperfine Raman transitions^39–42^. The phonon number detection on one of the ions (say ion 1) is then employed for simultaneous inference of the temperature of both ions.

In what follows, firstly we introduce the theoretical model and we show how to describe the ionic vibrational modes. We propose a joint estimation scheme, where a single probe state is used to estimate the temperatures of the two ions ($T_1$ and $T_2$) with a single projective measurement corresponding to the number of excitations in the first ion vibrational degree of freedom. In particular, we calculate the Fisher information as a function of the temperature of the ions and the interaction parameter *g*. We analyze the Fisher information of both ions in two scenarios—First, we consider the ions innitially in equilibrium with two individual thermal baths with distinct temperatures. Secondly, we investigate how a change in the vibrational state of one of the ion affects the performance of our approach. For that we consider a non-thermal bath in equilibrium with one of the ions, and also include the effect of spurious heating originating from the surface-electrode noise.

## Two interacting charges

**Figure 1:**
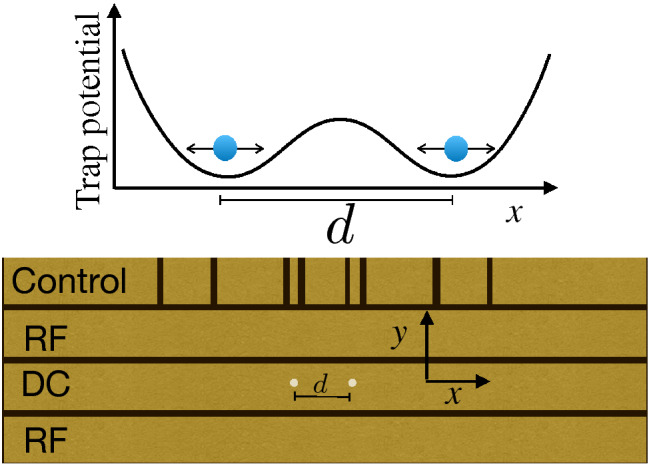
Scheme of two interacting trapped ions with the same charge *q*. Each ion is confined in a potential well along of coordinate *x* and separated by a distance *d*. Schematic diagram based on Ref.^37^, where the trap electrodes are placed in a single plane of a chip-based trap^46^. The RF and DC electrodes are placed to form a pseudopotential minimum above the surface, with the longitudinal confinement and multiple regions of confinement guaranteed by DC end caps, alternating with RF voltages, as depicted by the Control electrodes.

Ion trapping has a long and rich history, from its origins^43,44^, to the usage as a potential platform for quantum computation^25–29,45^. The ions can be confined in free space with oscillating electromagnetic fields supplied by nearby electrodes^45^, known as Paul Traps^44^. Originally constituted by hyperbolic electrodes, with time they were substituted, for more practical purposes, by surface-electrode Paul Traps^46^. Those are microfabricated planar linear traps, where a pseudo-potential allow ions to be held at distances of ~100 µm above the electrode surface. A typical configuration represented in Fig. 1, following the design in Ref.^37^, shows alternating DC and AC electrodes, which depending on the experimental purposes allow strong confinement in the transversal direction and controllable confinement along the longitudinal direction (*x* in this paper). This system was previously employed to trap and investigate the interaction of two ions^37,38^. Given the trapping conditions, after cooling, the motional degree of freedom of the ions can be seen as coupled quantized mechanical oscillators held in separate locations. The direct coupling, through the mutual Coulomb interaction of two ions, was used and probed. Controllable coupling between quantized mechanical oscillators is quite useful for investigation and simulation of several properties observed in Nature. In particular, in Ref.^37^, ${}^{9}$Be$^+$ ions are held 40 µm apart by trapping potentials (See Fig. 1), and are both Doppler-cooled to $T= 5.32\times 10^{-4}$ K (thermal average number of ${\bar{n}}=2.3$). After side-band cooling one of the ions to $T= 1.42\times 10^{-4}$ K (thermal average number of ${\bar{n}}=0.35$), its population is probed, observing asymmetry between the red and blue motional sidebands of hyperfine Raman transitions. This ion population shows a signature of the population exchange, and therefore the interaction between ions.

Here we focus on the detailed derivation and investigation of temperature inference for a similar situation to the one discussed in^37,38^. Let us consider two interacting trapped ions having the same charge *q* and mass *m*. Each ion is confined in a potential well along of coordinate *x* and separated by a distance *d*, as shown in Fig. 1, accordingly with refs.^37,38^.

Considering only the coordinates along the longitudinal separation of the ions, the Hamiltonian that describes this system reads^37,38^

3 $$\begin{aligned} H = \sum _{j=1}^2\left( \frac{p_{j}^{2}}{2m}+\frac{1}{2}m\omega ^{2}x_{j}^{2}\right) +H_{e}, \end{aligned}$$

where $\omega$ is the frequency of oscillation of each ion due to the trapping potential, and $p_i$ and ${x_i}$ are the momentum and position of the ion $i=1,2$, respectively. The term $H_{e}$ is the ions electrostatic interaction energy given by^47^

4 $$\begin{aligned} H_{e}&= \frac{1}{4\pi \epsilon _{0}}\frac{q^2}{d+ x_2 - x_1 }, \end{aligned}$$

where $(4\pi \epsilon _{0})^{-1}=9\times 10^{9} Nm^{2}/C^{2}$. By expanding (4) in powers of $\frac{x_{2} - x_{1}}{d}$, and considering small relative displacements we can rewrite the Coulomb interaction energy $H_{e}$ approximately as

5 $$\begin{aligned} H_{e}(d) \approx \frac{q^2}{4\pi \epsilon _{0}d}\left[ 1-\frac{x_{2} - x_{1}}{d}+\frac{\left( x_{2} - x_{1}\right) ^{2}}{d^{2}}\right] . \end{aligned}$$

Introducing the operators

6 $$\begin{aligned} x_{i}=\sqrt{\frac{ \hslash }{2m\omega }}(a_{i}^{\dagger } +a_{i}), i=1,2, \end{aligned}$$

and

7 $$\begin{aligned} p_{i}=i\sqrt{\frac{ \hslash m\omega }{2}}(a_{i}^{\dagger }-a_{i}), i=1,2, \end{aligned}$$

the total system Hamiltonian becomes

8 $$\begin{aligned} H&= \hslash \Omega a_{1}^{\dagger }a_{1}+ \hslash \Omega a_{2}^{\dagger }a_{2} +\sqrt{\frac{\hslash k^2 q^4}{2m\omega d^4}}(a_{1}+a_{1}^{\dagger })-\sqrt{\frac{\hslash k^2 q^4}{2m\omega d^4}} (a_{2}+a_{2}^{\dagger }) \nonumber \\&\quad+\frac{\hslash kq^2}{2m\omega d^{3}}(a_{1}^{2}+a_{1}^{\dagger 2})+\frac{\hslash k q^2}{2m\omega d^{3}}(a_2^{2}+a_{2}^{\dagger 2}) -\frac{\hslash k q^2}{m\omega d^{3}}(a_{1}a_{2}^{\dagger }+a_{2} a_{1}^{\dagger }+a_{1}a_{2}+a_{1}^{\dagger }a_{2}^{\dagger }), \end{aligned}$$

where $\Omega =\omega +(kq^2/m\omega d^3)$ are the ions trap frequencies deviated by a small frequency shift due the ions interaction, and $k=1/4\pi \epsilon _{0}$. In the interaction picture obtained by the unitary transformation $U=e^{-\frac{i}{\hslash }H_{o}t}$, where $H_{o}=\hslash \Omega a_{1}^{\dagger }a_{1}+ \hslash \Omega a_{2}^{\dagger }a_{2}$ corresponds to the trapped ions free evolution, the Hamiltonian becomes

9 $$\begin{aligned} {\tilde{H}}&= \hslash \sqrt{\frac{\hslash k^2 q^4}{2m\omega d^4}}(a_{1}e^{-i\Omega t}+a_{1}^{\dagger }e^{i\Omega t})-\sqrt{\frac{\hslash k^2 q^4}{2m\omega d^4}} (a_{2}e^{-i\Omega t}+a_{2}^{\dagger }e^{i\Omega t}) +\frac{\hslash kq^2}{2m\omega d^{3}}(a_{1}^{2}e^{-2i\Omega t}+a_{1}^{\dagger 2}e^{2i\Omega t})\nonumber \\&\quad+\frac{\hslash k q^2}{2m\omega d^{3}}(a_2^{2}e^{-2i\Omega t}+a_{2}^{\dagger 2}e^{2i\Omega t}) -\frac{\hslash k q^2}{m\omega d^{3}}(a_{1}a_{2}^{\dagger }+a_{2} a_{1}^{\dagger }+a_{1}a_{2}e^{-2i\Omega t}+a_{1}^{\dagger }a_{2}^{\dagger }e^{2i\Omega t}), \end{aligned}$$

and the terms oscillating at frequencies $\Omega$ and $2\Omega$ are averaged to zero for typical scales of time and can be disregard. The ions interaction Hamiltonian reduces to the simple form

10 $$\begin{aligned} {\tilde{H}}= \hslash g \left( a_{1}a_{2}^{\dagger }+a_{1}^{\dagger }a_{2}\right) , \end{aligned}$$

with the coupling constant $g = - kq^{2}/m\omega d^{3}$. It is important to note that the interaction (10) can be effectively controlled by detunig the individual ions motional frequencies, as discussed in Ref.^37^. Moreover, this coupling Hamiltonian is similar in form to that one for two radiation modes interacting through a beam-splitter. It is well known that a beam-splitter can only entangle two light modes if at least one of them is non-classical^48–50^. Therefore, it is interesting to investigate whether the non-classicality of one of the states affects the process of temperature inference. For that, in the next section, we develop a formalism that allows to tackle the dynamics of the two ions vibrational modes independently of the initial equilibrium conditions. Since we focus on the specific experimental conditions reported in Refs.^37,38^, it is interesting to fix some quantities used along the remainder of this paper, for the reader reference, as presented in Table 1. Moreover, squeezing in the vibrational mode of trapped ions was reported in several occasions, since^41,42^ (See Ref.^51^ for an excellent account on squeezing in the motional states of ions.). Particularly in Ref.^52^ it was reported a squeezing parameter as high as $r= 2.37$, which is well above the range of $0\le r\le 1.5$, employed here.

**Table 1:** Parameter used along this article, following experimental values employed in Ref.^37^.

$\Omega$	*d*	*g*	*T* (${\bar{n}}$)	$d\left\langle {\bar{n}}_i\right\rangle /dt=\Gamma , i=1,2$
$2\pi \times 4$ MHz	40 µm	$\pi \times 3.1$ kHz	$1.42 - 5.32 \times 10^{-4}{\rm K} \;\; (0.35-2.3)$	1.885 quanta/ms

From left to right: $\Omega$ is the ion vibrational angular frequency, *d* is the mean distance between ions, *g* is the ions coupling constant, *T* is the temperature with respective thermal average number ${\bar{n}}$ inside parenthesis, and $\Gamma$ is the heating rate due to surface-electrode noise.

## Bipartite Gaussian states

All states easily accessed experimentally for our purposes, are Gaussian. Therefore, here we develop the formalism more appropriate to investigate the properties of the ionic vibrational degrees of freedom. A two-mode bipartite quantum state $\rho$ is Gaussian if its symmetric characteristic function^53^ is given by $C({\varvec{\eta }})=Tr[D({\varvec{\eta }})\rho ]=e^{-\frac{1}{2}{\varvec{\eta }^\dagger }{\mathbf{V}}{\varvec{\eta }} }$, where $D(\varvec{\eta })=e^{-\varvec{\eta }^\dagger {{\mathbf {E}}}{{\varvec{v}}}}$ is a displacement operator in the four-vector $\varvec{\eta }$-space: $\varvec{\eta }^\dagger =\left( \eta _1^*, \eta _1, \eta _2^*, \eta _2\right)$, ${{\mathbf {v}}}^\dagger = \left( a_1^\dagger , a_1, a_2^\dagger , a_2\right)$, and

11 $$\begin{aligned} {\mathbf {E}}=\left( \begin{array}{c c}{{\mathbf {Z}}}&{}{\mathbf {0}}\\ {\mathbf {0}}&{} {{\mathbf {Z}}}\end{array}\right) ,\;\;\; {{\mathbf {Z}}}=\left( \begin{array}{c c}{1}&{}{0}\\ {0}&{} {-1}\end{array}\right) , \end{aligned}$$

where $a_1$ ($a_1^\dagger$) and $a_2$ ($a_2^\dagger$) are annihilation (creation) operators for party 1 and 2, respectively. ${{\mathbf {V}}}$ is a $4\times 4$ covariance matrix with elements $V_{ij}=(-1)^{i+j}\langle \{v_i,v_j^\dagger \}\rangle /2$, which can be decomposed in four block $2\times 2$ matrices,

12 $$\begin{aligned} {{\mathbf {V}}}=\left( \begin{array}{c c}{{\mathbf {V}}_1}&{}{\mathbf{C}}\\ {{\mathbf {C}}}^\dagger &{} {{\mathbf {V}}_2}\end{array}\right) , \end{aligned}$$

where ${{\mathbf {V}}_1}$ and ${{\mathbf {V}}_2}$ are $2\times 2$ Hermitian matrices containing only local elements, while ${{\mathbf {C}}}$ is a $2\times 2$ matrix representing the correlation between the two parties and explicitly are written as

13 $$\begin{aligned} \mathbf { V_i}=\left( \begin{array}{c c} n_i&{}m_i\\ m_i^*&{} n_i\end{array}\right) , i=1,2, \;\;\; {{\mathbf {C}}}=\left( \begin{array}{c c}{m_s}&{}{m_c}\\ {m_c^*}&{} {m_s^*}\end{array}\right) . \end{aligned}$$

Positivity and separability for bipartite Gaussian quantum states have been largely investigated^54–56^. Besides the requirement of the uncertainty principle

14 $$\begin{aligned} {{\mathbf {V}}}+\frac{1}{2} {{\mathbf {E}}}\ge 0, \end{aligned}$$

there is a necessary and sufficient condition, which must be satisfied for separable Gaussian states^56^

15 $$\begin{aligned} \mathbf {{\widetilde{V}}}+\frac{1}{2} {\mathbf {E}}\ge 0, \end{aligned}$$

under a partial phase space mirror reflection, $\mathbf {\widetilde{V}}=\mathbf {TVT}:{{\mathbf {T}} {\mathbf {v}}^\dagger }={\mathbf{v}^\dagger }{\mathbf {T}}= \left( a_1^\dagger , a_1,a_2,a_2^\dagger \right)$, with

16 $$\begin{aligned} {{\mathbf {T}}}=\left( \begin{array}{c c}{{\mathbf {I}}}&{}{\mathbf{0}}\\ {{\mathbf {0}}}&{} {{\mathbf {X}}}\end{array}\right) ,\, \text {and} \,\, {{\mathbf {X}}}=\left( \begin{array}{c c}{ 0}&{}{ 1}\\ {1}&{} {0}\end{array}\right) . \end{aligned}$$

The physical positivity criterion (14) applies only if^56^

17 $$\begin{aligned} n_1&\ge \sqrt{|m_1|^2+\frac{1}{4}}, \end{aligned}$$

18 $$\begin{aligned} n_2&\ge \frac{s }{d }+\sqrt{\frac{1}{4} \left[ \frac{\left| |m_c|^2-|m_s|^2\right| }{d }-1\right] ^2+|m_2-c |^2}, \end{aligned}$$

respectively, with $s =n_1\left( |m_c|^2+|m_s|^2\right) -m_cm_sm_1^*-m_c^*m_s^*m_1$, $c =2n_1m_s^*m_c-m_c^2m_1^*-(m_s^*)^2m_1$, and $d =n_1^2-\frac{1}{4}-|m_1|^2$. Similarly the separability condition (15) writes explicitly into (17) and

19 $$\begin{aligned} n_2&\ge \frac{s }{d }+\sqrt{\frac{1}{4} \left[ \frac{\left| |m_c|^2-|m_s|^2\right| }{d }+1\right] ^2+|m_2-c |^2}. \end{aligned}$$

The ions vibrational modes are assumed as initially uncoupled, so that in (12) ${\mathbf {C}}={\mathbf {0}}$, and they are prepared in special local states to be discussed later. However, the effect of the interaction (10) is to correlate the two modes. This can be seen as the following Bogoliubov operation *B*:

20 $$\begin{aligned} \rho _{out}&= B\rho _{in}B^\dagger , \end{aligned}$$

21 $$\begin{aligned} B{{\mathbf {v}}}B^\dagger&= {{\mathbf {M}}} {{\mathbf {v}}}, \end{aligned}$$

22 $$\begin{aligned} {{\mathbf {M}}}&= \left( \begin{array}{c c}{{\mathbf {R}}}&{}{\mathbf{S}}\\ {-{\mathbf {S}}}^*&{} {{\mathbf {R}}}^*\end{array}\right) , {\mathbf{R}}=\cos \theta \left( \begin{array}{cc} e^{i\phi _0}&{}0\\ 0&{} e^{-i\phi _0}\end{array}\right) ,{\mathbf{S}}=\sin \theta \left( \begin{array}{cc}e^{i\phi _1}&{}0\\ 0&{}e^{-i\phi _1}\end{array}\right) , \end{aligned}$$

where $\rho _{out}$ is the density operator for the joint output state. The output symmetric characteristic function is given by

23 $$\begin{aligned} C_{out}({\varvec{\eta }})=Tr[D({\varvec{\eta }})\rho _{out}]=Tr[B^\dagger D({\varvec{\eta }})B\rho _{in}]. \end{aligned}$$

Now with the help of Eqs. (11–13), $B^\dagger D({\varvec{\eta }})B=e^{-\varvec{\eta }^\dagger {\mathbf{E}}{{\mathbf {M}}}^{-1}{{\mathbf {v}}}}\equiv D({\mathbf {\zeta }})$, with $\mathbf {\zeta }={{\mathbf {M}}}\varvec{\eta }$, since ${{\mathbf {M}}} {{\mathbf {E}} M}^{-1}={{\mathbf {E}}}$. Thus

24 $$\begin{aligned} C_{out}({\varvec{\eta }})=C_{in}({\mathbf {\zeta }})=e^{-\frac{1}{2}{\mathbf {\zeta }^{\dagger }}{\mathbf{V}}{\mathbf {\zeta }} }=e^{-\frac{1}{2}{\varvec{\eta }^{\dagger }}{\mathbf{V}^\prime }{\varvec{\eta }} }, \end{aligned}$$

where ${\mathbf{V}}^\prime ={{\mathbf {M}}}^{-1}{{\mathbf {V}}}{{\mathbf {M}}}$, and analogously to (12), ${{\mathbf {V}}}^\prime$ can be block decomposed with

25 $$\begin{aligned} {{\mathbf {V}}_1^\prime }&= {{\mathbf {R}}}^{*}{{\mathbf {V}}_1}{\mathbf{R}}+{\mathbf{S}}{{\mathbf {V}}_2}{{\mathbf {S}}}^*, \end{aligned}$$

26 $$\begin{aligned} {{\mathbf {V}}_2^\prime }&= {\mathbf{S}}^{*}{{\mathbf {V}}_1}{{\mathbf {S}}}+{{\mathbf {R}}}{{\mathbf {V}}_2}{{\mathbf {R}}}^{*}, \end{aligned}$$

27 $$\begin{aligned} {{\mathbf {C}}^\prime }&= {{\mathbf {R}}}^{*}{{\mathbf {V}}_1}{\mathbf{S}}-{{\mathbf {S}}}{{\mathbf {V}}_2}{{\mathbf {R}}}^{*}. \end{aligned}$$

For the specific unitary transformation induced by the time-independent two-modes coupling (10) $\phi _0=\phi _1=0$, and $\theta = g t$, where *t* is the time variable. Typically, the separability criterion (15) is recast in terms of the local symplectic invariants, belonging to the $Sp(2,R) \otimes Sp(2,R)$ group, $I_1=\det \mathbf {V_1}$, $I_2=\det \mathbf {V_2}$, $I_3=\det {\mathbf {C}}$, and $I_4=\text {tr}(\mathbf {V_1}{\mathbf {Z}}{\mathbf {C}}{\mathbf {Z}} {\mathbf {V}}_2{\mathbf {Z}}\mathbf {C^\dagger }{\mathbf {Z}})$. Using the $I_1=\det \mathbf {V_1}$, $I_2=\det \mathbf {V_2}$, $I_3=\det {\mathbf {C}}$, and $I_4=\text {tr}(\mathbf {V_1}{\mathbf {Z}}{\mathbf {C}}{\mathbf {Z}} {\mathbf {V}}_2{\mathbf {Z}}\mathbf {C^\dagger }{\mathbf {Z}})$. A state is not entangled if, and only if^55^

28 $$\begin{aligned} I_1I_2 + \left( 1/4 - |I_3|\right) ^2 - I_4 - (I_1 + I_2)/4\ge 0. \end{aligned}$$

We will employ the violation of the bound (28) as an entanglement indicator, after we define, the initial motional states.

As mentioned before, we shall consider the case where the ions are initially uncorrelated so the total density operator factorizes as

29 $$\begin{aligned} \rho _{12}= \rho _{1}\otimes \rho _{2}, \end{aligned}$$

where $\rho _1$ and $\rho _2$ are the reduced density operators of ions 1 and 2, respectively. We assume that the ion 1 is initially in equilibrium with a thermal reservoir at temperature $T_1$, while ion 2 can be in equilibrium with a thermal or non-thermal (for future purposes) reservoir at temperature $T_2$. For that, we introduce a controllable squeezing parameter so that the ion 2 can be prepared in a thermal squeezed state. Thus, the reduced density operator for the ions 1 and 2 in a coherent state representation are written as

30 $$\begin{aligned} \rho _1 = \frac{1}{\pi {\bar{n}}_1}\int d^{2}\alpha {e^{-\frac{|\alpha |^2}{{\bar{n}}_1}}} | \alpha \rangle \langle \alpha |, \end{aligned}$$

and

31 $$\begin{aligned} \rho _2 = \frac{1}{\pi {\bar{n}}_2}\int d^{2}\beta {e^{-\frac{|\beta |^2}{{\bar{n}}_2}}} S | \beta \rangle \langle \beta | S^{\dagger }, \end{aligned}$$

respectively, where

$$\begin{aligned} S&= e^{\frac{r}{2}\left( \xi ^{*}a_{2}^{2}-\xi a_{2}^{\dag 2}\right) } \end{aligned}$$

is the one-mode squeezing operator, $\xi = e^{i2\phi }$, and the average thermal photon numbers are given by

32 $$\begin{aligned} {\bar{n}}_1 = \frac{1}{e^{\frac{\hslash \Omega }{k_{B}T_1}} - 1},\quad {\bar{n}}_2 = \frac{1}{e^{\frac{\hslash \Omega }{k_{B}T_2}} - 1}, \end{aligned}$$

where $T_1$ and $T_2$ correspond respectively to the equilibrium temperature of the ion-1 and ion-2.

The time evolution of the density operator with Hamiltonian (10) occurs with the following expression

33 $$\begin{aligned} \rho _{12}(\theta ) = U(\theta )\rho _{12}U^{\dagger }(\theta ), \end{aligned}$$

where $$\theta =gt$$, and

34 $$\begin{aligned} U(\theta ) = \exp [-i\theta (a_{1}a_{2}^{\dagger } + a_{1}^{\dagger }a_{2})]. \end{aligned}$$

**Figure 2:**
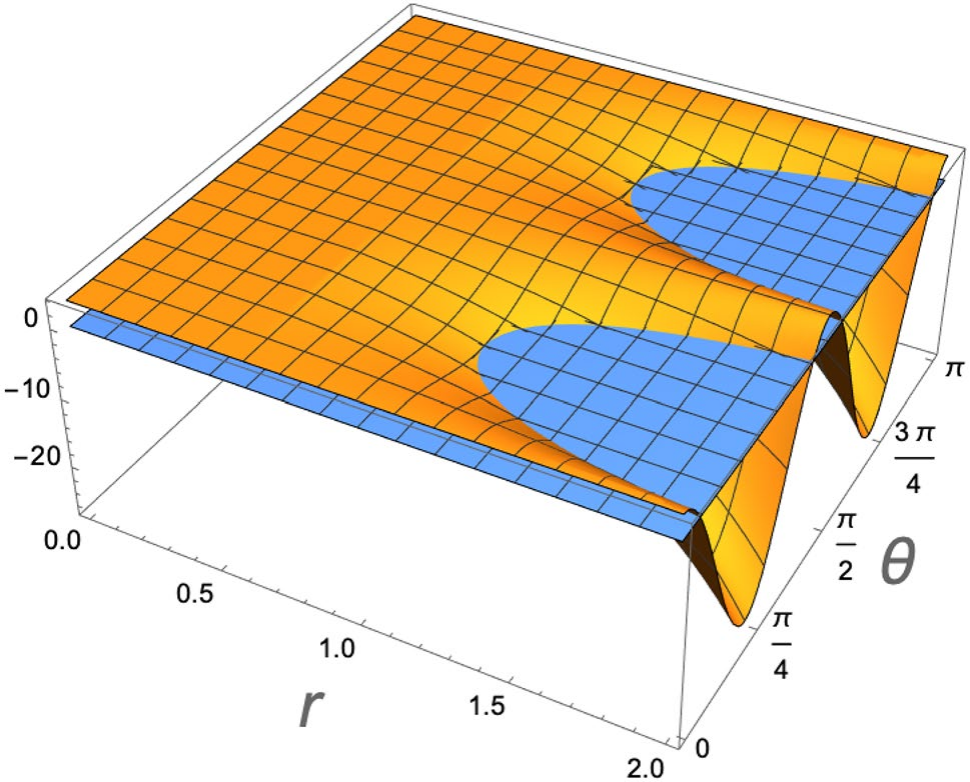
Entanglement of the ions logitudinal vibrational modes as a function of the squeezing parameter of ion 2, and the interaction $\theta =gt$. Only for $2({\bar{n}}_{2}+1/2)\le e^{2r}$, is that the vibrational mode 2 is non-classical, allowing that the evolution (34) entangle the ions (represented by the negative values of the vertical axis). Maximal entanglement occurs at $\theta =\pi /4$ (or $3\pi /4$), corresponding to a 50:50 beam-splitter.

To understand the correlation induced by (34), we plot in Fig. 2 the bound (28) as a function of $\theta$ and the squeezing parameter *r* for the ion 2, for a fixed temperature of $T_{1}=1.42\times 10^{-4}$ K (${\bar{n}}_1=0.35$), and $T_2=5.32\times 10^{-4}$ K (${\bar{n}}_2=2.3$). Only for $2({\bar{n}}_{2}+1/2)\le e^{2r}$, is that the vibrational mode 2 is non-classical, being a necessary (but not sufficient^56^) condition for the evolution (34) to entangle the vibrational modes of the ions. As it is expected, the maximal entanglement occurs at $\theta =\pi /4$ (or $3\pi /4$), which corresponds to a 50:50 beam-splitter. Only for $r\ge 1.1$ is that the system can be entangled.

## Fisher information

**Figure 3:**
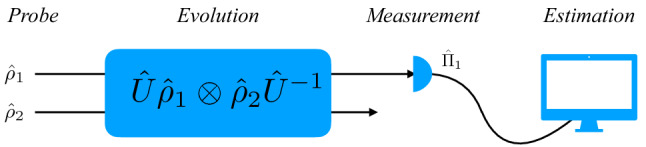
Scheme for the indirect estimation of the temperatures of two interacting trapped ions. After some interaction time between the two ions, a energy measurement corresponding to the number of phonons is performed on the 1-ion, followed by post-processing for the estimation of parameters $T_1$ and $T_2$.

The trapping conditions require several steps of cooling and electromagnetic confinement of ions. Typically, the ions coupling parameter *g* is very small ($g=\pi \times 3.1$ kHz, here), and therefore the temperature of the system must reach cryostatic regimes of a few hundred microkelvins. Therefore, an inhomogeneity is expected and the individual ions equilibrium temperatures may differ. Further experimental limitations constrains the direct access to all ions in the trap^37^, as was suggested by the inference approach^24^. Our purpose here is to use the phonon number detection (over one of the ions only) as a way to infer simultaneously the temperature differences of both ions. In particular, the estimation of the temperatures of the ions can be implemented by choosing the projective measurement corresponding to the number of phonons in the longitudinal vibrational mode of the ion 1, as described in Fig. 3. It shows our estimation scheme, where the dynamics of two trapped ions is governed by the unitary operation (34).

In the temperature estimation procedure, it is essential to have access to the ion 1 longitudinal vibrational mean number. This is a standard experimental detection procedure, well described in the literature^39,51^. An individual ion vibrational mode phonon number is accessed experimentally, through the observation of the asymmetry between the red and blue motional sidebands of hyperfine Raman transitions^39^. For ${}^{9}$Be$^+$ ions, for example, during the Raman cooling, the internal $|\downarrow \rangle =|F=2,m_F=-2\rangle$ and $|\uparrow \rangle =|F=1,m_F=-1\rangle$ hyperfine states are accessed by two counter-propagating Raman beams. In this process, transitions from $|\downarrow \rangle |n\rangle$ to $|\uparrow \rangle |n-1\rangle$ (where $|n\rangle$ is the vibrational state of the ion) are induced cyclically, together with dissipative repumping, resulting in the cooling of the ion 1. It also allows to access the probabilities of measuring $|\downarrow \rangle$, $P_{\downarrow }^{RSB}$ and $P_{\downarrow }^{BSB}$, after excitation with red or blue sideband, respectively^39,51^. The average motional occupation is given by ${\bar{n}}_1=R/(1-R)$, where $R=P_{\downarrow }^{RSB}/P_{\downarrow }^{BSB}$. In that fashion the population of the vibrational state of ion 1 is measured. We can calculate the Fisher information corresponding to the parameters $T_1$ and $T_2$ via the number of excitations ${\hat{\Pi }}_1 = \left| k\right\rangle \left\langle k\right|$ in the longitudinal vibrational degree of freedom of ion 1.

For the number of phonon measurements, the Fisher information for ion 1 and ion 2 is computed as

35 $$\begin{aligned} F_{\alpha \beta } = \sum _{k} P_{1}(k) \left( \frac{\partial \ln P_{1}(k)}{\partial T_\alpha } \right) \left( \frac{\partial \ln P_{1}(k)}{\partial T_\beta } \right) ,\,\,\,\,\,\,\,\, \alpha ,\beta = 1,2 \end{aligned}$$

where $P_1(k)$ is the probability distribution of the occupation number obtained by measuring the energy of ion 1.

**Figure 4:**
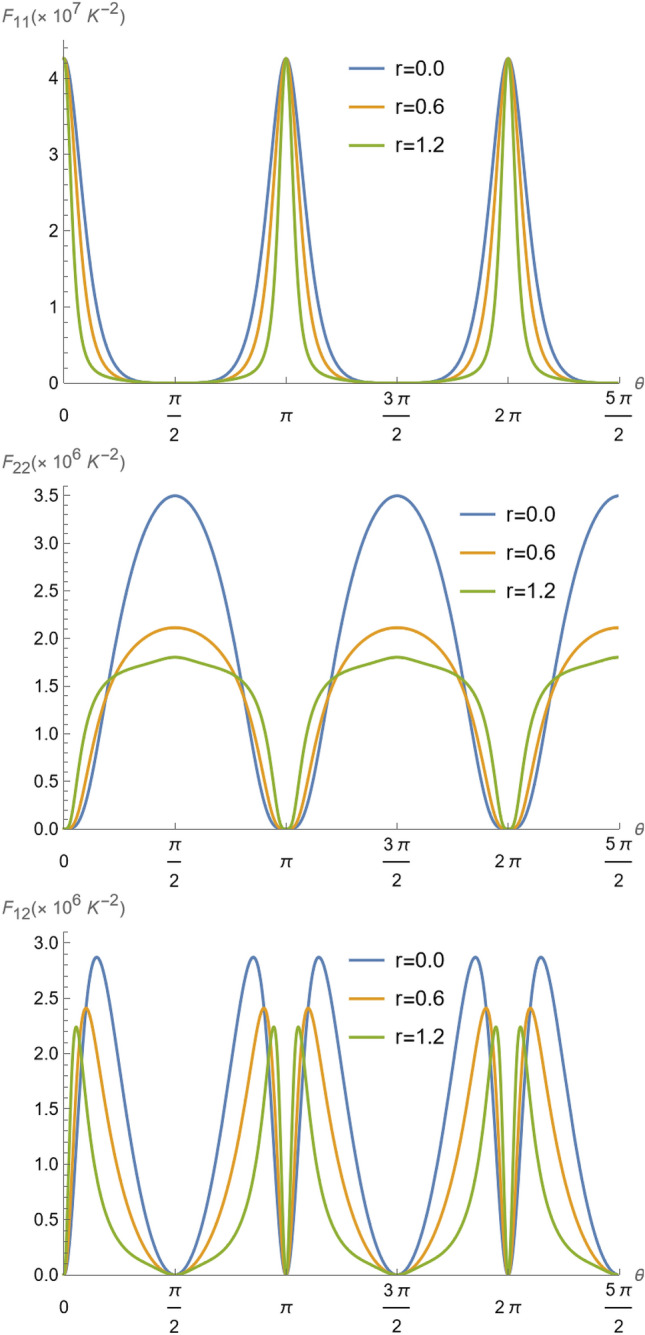
Fisher information $F_{11}$, $F_{22}$ and $F_{12}$ as a function of parameter $\theta =gt$, for $T_{1}=1.42\times 10^{-4}K$ (${\bar{n}}_{1} = 0.35$), $T_{2}=5.32\times 10^{-4}K$ (${\bar{n}}_{2} =2.3$), and $\Omega /2\pi = 4$ MHz^37,57^.

**Figure 5:**
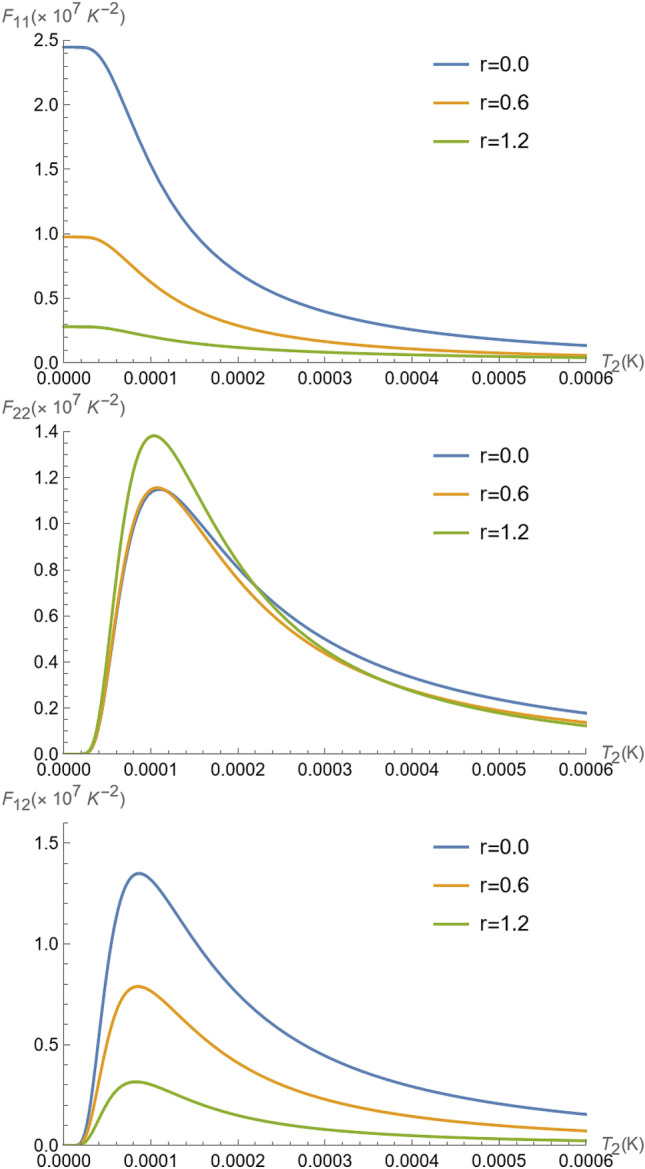
Fisher information $F_{11}$, $F_{22}$ and $F_{12}$ as a function of parameter $T_{2}$ for $\theta =\pi /4$, $T_{1}=1.42\times 10^{-4}K$ (${\bar{n}}_1=0.35$), and $\Omega /2\pi = 4$ MHz^37,57^.

The probability distribution of *k* phonons is given by

36 $$\begin{aligned} P_{1}(k)=\frac{1}{k!\sqrt{\det ({{\mathbf {V}}_{1}'}-\frac{1}{2}{\mathbf{I}})}}\int d^{2}\gamma \,\, |\gamma |^{2k} e^{\frac{1}{2}\gamma ^{\dagger } {{\mathbf {Z}}}({\mathbf{V}_{1}'}-\frac{1}{2}{{\mathbf {I}}})^{-1}{{\mathbf {Z}}}\gamma -|\gamma |^2 } , \end{aligned}$$

where

37 $$\begin{aligned} {{\mathbf {V}}}_{1}'-\frac{1}{2}{{\mathbf {I}}}=\begin{pmatrix} n'_1 &{} m'_1 \\ m'^{*}_1 &{} n'_1 \end{pmatrix}, \end{aligned}$$

with

38 $$\begin{aligned} n'_1&= {\bar{n}}_{1}\cos ^2 {(gt)} +\left[ \left( {\bar{n}}_2+1/2\right) \cosh {2r}-1/2\right] \sin ^2 {(gt)}, \end{aligned}$$

39 $$\begin{aligned} m'_1&= \left( {\bar{n}}_2 +1/2\right) e^{2i\phi }\sinh {(2r)} \sin ^2 {(gt)}. \end{aligned}$$

The probability distribution given by Eq. (36) involves a Gaussian integral that can be evaluated exactly so that the probability distribution is given as follows

40 $$\begin{aligned} P_{1}(k)=\frac{(-1)^{k}}{k!\sqrt{{n'_1}^2-|m'_1|^2}}\frac{\partial ^{k}}{\partial A^{k}} \left( \frac{1}{\sqrt{A^2-|B|^2}}\right) , \end{aligned}$$

where

41 $$\begin{aligned} A^2=\left( 1+\frac{n'_1}{{n'_1}^2-|m'_1|^2}\right) ^2, \end{aligned}$$

and

42 $$\begin{aligned} |B|^2=\frac{|m'_1|^2}{\left( {n'_1}^2-|m'_1|^2\right) ^2}. \end{aligned}$$

The probability distribution does not depend on the squeezing phase since it willl not appear in the term $|m'_1|^2$.

Now using the series expansion

43 $$\begin{aligned} \frac{1}{\sqrt{A^2-|B|^2}}=\frac{1}{A}\sum _{n=0}^{\infty }\frac{(2n)!}{2^{2n}(n!)^2}\left( \frac{|B|}{A} \right) ^{2n}, \end{aligned}$$

the derivatives in relation to *A* are easily evaluated, so the probability distribution reduces to

44 $$\begin{aligned} P_{1}(k)=\frac{1}{A^{k+1}\, \sqrt{n_1'^2-|m_1'|^2}}\sum _{n=0}^{\infty }\frac{(2n+k)!}{k!(2^{n}n!)^2}\left( \frac{|B|}{A} \right) ^{2n}. \end{aligned}$$

The series in Eq. (44) can be identified with the Hypergeometric^58^ function $_{2} F_{1} \left( \frac{1+k}{2},\frac{2+k}{2},1,\left( \frac{|B|}{A}\right) ^2\right)$ so we can write

45 $$\begin{aligned} P_{1}(k)= \frac{ _2 F_{1} \left( \frac{1+k}{2},\frac{2+k}{2},1,\left( \frac{|B|}{A}\right) ^2\right) }{A^{k+1}\sqrt{n_1'^2-|m_1'|^2}} . \end{aligned}$$

In the case that $r=0$, with both modes in thermal states, the probability distribution reduces to the simple expression

46 $$\begin{aligned} P_{1}(k) = \frac{\left[ {\overline{n}}_{1}\cos ^2{(gt)}+ {\overline{n}}_{2}\sin ^2{(gt)}\right] ^k}{\left[ 1+{\overline{n}}_{1}\cos ^2{(gt)}+ {\overline{n}}_{2}\sin ^2{(gt)}\right] ^{k+1}}, \end{aligned}$$

and the Fisher information elements of Eq. (35) are obtained in a closed form given by

47 $$\begin{aligned} F_{\alpha \beta } =\frac{(\hslash \Omega /k_{B})^2}{T_{\alpha }^2 T_{\beta }^2} \frac{{\overline{n}}_{\alpha }{\overline{n}}_{\beta }(1+{\overline{n}}_{\alpha })(1+{\overline{n}}_{\beta }) \cos ^2{(gt+\frac{\pi }{2}\delta _{\alpha 2})} \sin ^2{(gt+\frac{\pi }{2}\delta _{1 \beta })} }{\left[ {\overline{n}}_{1}\cos ^2{(gt)}+ {\overline{n}}_{2}\sin ^2{(gt)}\right] \left[ 1+{\overline{n}}_{1}\cos ^2{(gt)}+ {\overline{n}}_{2}\sin ^2{(gt)}\right] },\,\,\,\alpha , \beta = 1,2 \end{aligned}$$

where $\delta _{\alpha \beta }$ is the Kronecker delta. To obtain the values of the Fisher information for non null squeezing ($r\ne 0$) we set the sums up to $k=200$ in Eqs. (35) in order to guarantee convergence in the numerical calculations.

In Fig. 4, the Fisher information $F_{11}$, $F_{22}$ and $F_{12}$ evolution with $\theta =gt$, is represented for two thermal reservoirs (for $r=0$) at $T_{1}=1.42\times 10^{-4}K$, and $T_{2}=5.32\times 10^{-4}K$, corresponding respectively to the experimental values of ${\bar{n}}_{1}=0.35$ and ${\bar{n}}_{2}=2.3$ in Ref.^37^, and for a non-thermal reservoir (for $r\ne 0$). We can see by the corresponding curves of $F_{11}$ that the temperature $T_1$ is best inferred at the instants where $\theta =n\pi$, for $n=0,1,2,\ldots$. This occurs because at those instants the initial equilibrium state of the ion 1 has recurred^59^. In contrast, as can be seen in $F_{22}$, at $\theta = m \frac{\pi }{2}$, for $m= 1, 3, 5,\ldots$ the state of ions 1 and 2 has been swapped, by the characteristic of evolution (34), and therefore it is not surprising that those are the best instants for inference of temperature of the vibrational mode of ion 2—it occurs because when the states are interchanged^59^, the detection of the ion 1 vibrational mode is, in fact, the detection over the state of ion 2. Simultaneous inference of both ions temperatures occurs when $\theta = m \frac{\pi }{4}$, for $m= 1, 3, 5,\ldots$, as depicted by $F_{12}$. Even when a non-thermal state is involved, the approach is successful for the inference of the ion 2 temperature, as we can see in the same figures by varying *r*, the same characteristics hold. The effect of squeezing, however, is to improve the entanglement, and therefore it turns out that the distribution of points where the inference is optimal is spread, meaning that there is a slight advantage in using entanglement as a way to improve the accessibility at distinct instants of time for inference.

**Figure 6:**
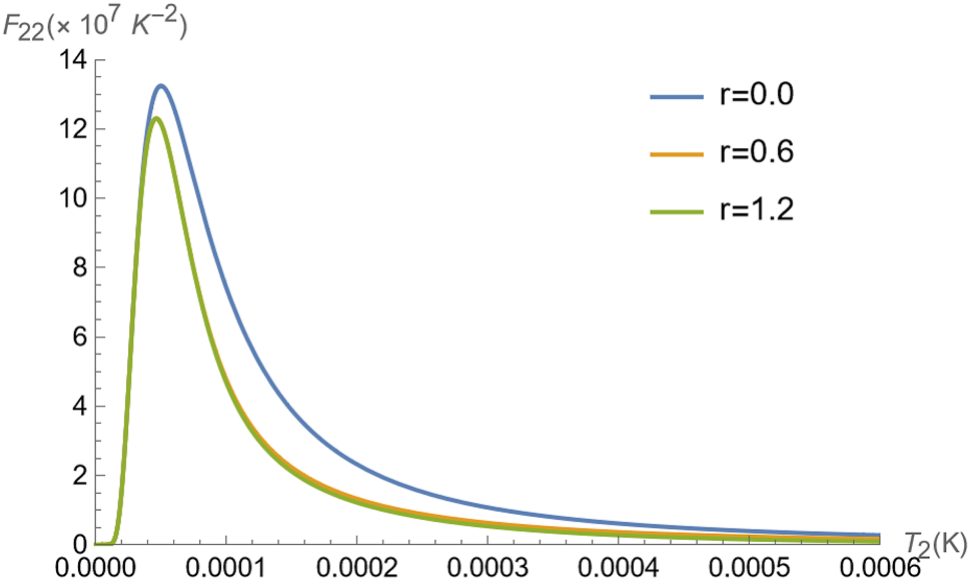
Fisher information $F_{22}$ as a function of parameter $T_{2}$ for $\theta =\pi /2$, and $\Omega /2\pi = 4$ MHz^37,57^. At $\theta =\pi /2$, all Fisher information becomes independent of $T_{1}$ since the ions have exchanged their states, and particularly, $F_{11}=F_{12}=0$.

In Fig. 5, the Fisher information $F_{11}$, $F_{22}$ and $F_{12}$ are depicted as functions of the temperature $T_{2}$ for $\theta =\pi /4$ (similar results are observed for odd multiples of $\theta =\pi /4$), and different values of *r*, for a fixed temperature of ion 1 at $T_{1}=1.42\times 10^{-4}K$. This result indicates a precision loss for the estimation of temperature of the ion 1, when we increase the *r* (this is reproduced in the graphs of $F_{11}$ and $F_{12}$ in Fig. 5. The same cannot be said for estimating only the temperature $T_{2}$, which is improved with an increase in *r* (see graphs of $F_{22}$ in Fig. 5) under the same conditions, working as a super probe in the non-classical regime $2({\bar{n}}_{2}+1/2)\le e^{2r}$, for this maximally entangled situation for $\theta =\pi /4$. In fact, the maximal precision is still observed at $\theta =\pi /2$, as depicted in Fig. 6, in contrast to $F_{11}$ and $F_{12}$, which are null for any $T_2$ we see that $F_{22}$ reaches optimal values, which however is disturbed by the non-thermal reservoir feature.

Lastly, we remark on the observed flat response regions observed for all $F_{\alpha \beta }, \alpha ,\beta =1,2$, in Figs. 5 and 6, for very low temperatures. To understand the reason behind that, we expand the exact expression for $F_{\alpha \beta }$ for $r=0$, from Eq. (47) for $T_{2}\rightarrow 0$ at $\theta =\pi /4$. Thus, at very low $T_2$ the Fisher information elements behave as

48 $$\begin{aligned} F_{11}\approx d_{11}-c_{11} e^{-\hslash \Omega /k_BT_2},\;\; F_{22}\approx c_{22}\frac{e^{-2\hslash \Omega /k_BT_2}}{T_{2}^4},\;\; F_{12}\approx c_{12} \frac{e^{-\hslash \Omega /k_BT_2}}{T_{2}^2}, \end{aligned}$$

where $d_{11}$ and $c_{\alpha ,\beta }$, with $\alpha ,\beta =1,2$, are constants dependent on the other parameters in(47). Since the exponentials rapidly decrease as $T_2$ goes to zero, it becomes clear the plateau in the Fisher information elements in Figs. 5 and 6 for small values of $T_2$. The physical reason is that at small $T_2$, the vibrational energy of ion 1 is not sufficient to disturb the inference information as given by the $F_{\alpha \beta }$. This is because the quantum state of the vibrational degree of motion of ion 1 is not considerably disturbed, and therefore in $F_{11}$ the dispersion in the measurement of the average number of phonons of ion 1 is dependent only on the quantum statistical properties of its state. This is reinforced by the fact that $F_{22}$ and $F_{12}$ remain close to zero at the same interval that the plateau occurs in $F_{11}$, meaning that no information about the vibrational energy of ion 2 can be obtained.

## Heating due to surface-electrode noise

Fluctuations in the trap parameters may introduce perturbations in the equilibrium state of each distinct ion as we discussed in the previous section. In addition, a thermal heating rate due noisy electrical potentials on the trap electrode surfaces is typically present (For an excellent account, please see Ref.^46^). The residual fluctuation in the electric fields couple to the motional degrees of freedom of the ions, inducing transitions between vibrational state. In^37^, e.g., a heating of 1.885 quanta per milisecond was observed, while in^38^ it was of 1.3 quanta per milisecond. The heating effect has been successfully treated through a master equation formalism^46^, or equivalently through the Heisenberg-Langevin equation formalism, as in Ref.^60^. In any case, the individual ions can be considered in thermal equilibrium with their individual reservoirs, whose temperatures are steadily increasing (in Thermodynamics it is formally stated that the system follows an equilibrium path, being sequentially in contact and in thermal equilibrium with an infinite number of reservoirs, each one with an infinitesimally different and increasing temperature.) and then suffering the individual heating at the same rate. This was the procedure successfully considered in^37,38^ to describe the heating rate observed experimentally. The Langevin approach turns out to effectively disturb the individual equilibrium bosonic operators for the vibrational degrees of freedom in the following form,

49 $$\begin{aligned} \frac{d a_i}{dt}&= -\frac{i}{\hslash }[a_i,H]-\frac{\gamma }{2} a_i+f_i(t)\nonumber \\&= -i(\Omega -i\frac{\gamma }{2}) a_i-ig a_j+f_i(t), i\ne j=1,2 \end{aligned}$$

where $f_i(t)$ are noise operators satisfying

50 $$\begin{aligned} \langle f_{i}(t)\rangle&= 0, \end{aligned}$$

51 $$\begin{aligned} \langle f_i^\dagger (t_1)f_j(t_2)\rangle&= \bar{N}\gamma \;\; \delta _{i,j}\;\delta (t_1-t_2), \end{aligned}$$

52 $$\begin{aligned} \langle f_i(t_1)f_j^\dagger (t_2)\rangle&= (\bar{N}+1)\gamma \;\; \delta _{i,j}\;\delta (t_1-t_2), \end{aligned}$$

53 $$\begin{aligned} \langle f_i(t_1)f_j(t_2)\rangle&= \langle f_i^\dagger (t_1)f_j^\dagger (t_2)\rangle =0, \end{aligned}$$

being $\gamma$ the decay constant induced by the effective memoryless reservoirs, with mean occupation number $\bar{N}$, modeling the effects of surface-electrode noise. We have assumed the same decay constant for both the ions, in order to simplify the analysis.

The solution of the system of coupled equations (49) can be obtained in terms of the noise, and modes operators at $t=0$ as

54 $$\begin{aligned} a_j(t)&= e^{-(i\Omega +\frac{\gamma }{2}) t}\left[ a_{j}(0)\cos {(gt)}-ia_{l}(0)\sin {(gt)} \right] + \int _{0}^{t}e^{-(i\Omega +\frac{\gamma }{2})(t-t')}\cos {[g(t-t')]} f_j (t') dt' \nonumber \\&-i\int _{0}^{t}e^{-(i\Omega +\frac{\gamma }{2})(t-t')}\sin {[g(t-t')]} f_l (t') dt', j\ne l=1,2, \end{aligned}$$

which, in turn, can be used to evaluate all the elements of the covariance matrix in Eq. (29). For the ion 1, using the properties of the noise operators, from Eqs. (50)–(53), we have

55 $$\begin{aligned} n_{1}(t)=e^{ -\gamma t}\left[ n_{1}(0)\cos ^2(gt) + n_{2}(0)\sin ^{2}(gt) \right] +\bar{N}\left( 1-e^{-\gamma t} \right) . \end{aligned}$$

Now, Eq. (55) clearly shows that, with time, the exchange of excitations between the vibrational modes of the ions ceases to exist and $n_1(t\rightarrow \infty )=\bar{N}$, reaching equilibrium with the effective reservoir. However, this timescale is unrealistic and too far from actual experimental conditions^46^, since the effective reservoir emulates the effect of spurious surface-electrode noise. Here we are interested in timescales consistent with the experimental conditions of Ref.^37^, where the damping in the excitations exchange is negligible. In addition, the damping mechanism introduced also takes into account the decoherence, through the decay of the other correlations appearing in Eqs. (25–27). In this timescale, where the decay is very slow compared to the excitation exchange timescale between ions ($\gamma \ll g$), we can approximate the exponential decay to its first relevant order in each term in Eq. (55) to get

56 $$\begin{aligned} n_1(t) \approx n_{1}(0)\cos ^{2}(gt) + n_{2}(0)\sin ^{2}(gt) +\bar{N}\gamma \, t, \end{aligned}$$

and the effective effect of noise is to introduce a linearly time dependent heating at rate $\Gamma = \bar{N}\gamma$ in the system, as experimentally observed in Ref.^37^. The same consideration, is obviously valid for mode 2. At the same timescale considered, the correlations are not affected by the heating noise, and therefore the motional state is not affected by decoherence. Other decoherence mechanisms, such as phase noise, may appear at a near timescale of a few miliseconds, particularly in experiments involving coupling between the motional and internal electronic states. In Refs.^61,62^, e.g., the observed decoherence time due to dephasing is around 5 ms. However, this is about 6.3 times larger than the timescale of $\theta =gt=5\pi /2$, corresponding to $t\approx 0.8$ ms, of the actual experimental condition of Ref.^37^. Therefore, at the timescale we are considering, decoherence does not take place, although the entanglement between the two vibrational modes is affected, since the global state becomes more classical with the heating, as we will discuss latter on. Again, at larger timescales the heating would be the dominant effect, and would already compromise the present strategy. In any case, when the internal electronic states are considered, one should employ a combined strategy for investigation of decoherence and heating, as in ref^61^, for example.

Therefore, Eq. (38), now reads

57 $$\begin{aligned} n'_1&= ({\bar{n}}_{1}+\Gamma t)\cos ^{2} {(gt)} +\left[ \left( {\bar{n}}_{2}+1/2\right) \cosh {2r}-1/2+\Gamma t\right] \sin ^{2} {(gt)}. \end{aligned}$$

**Figure 7:**
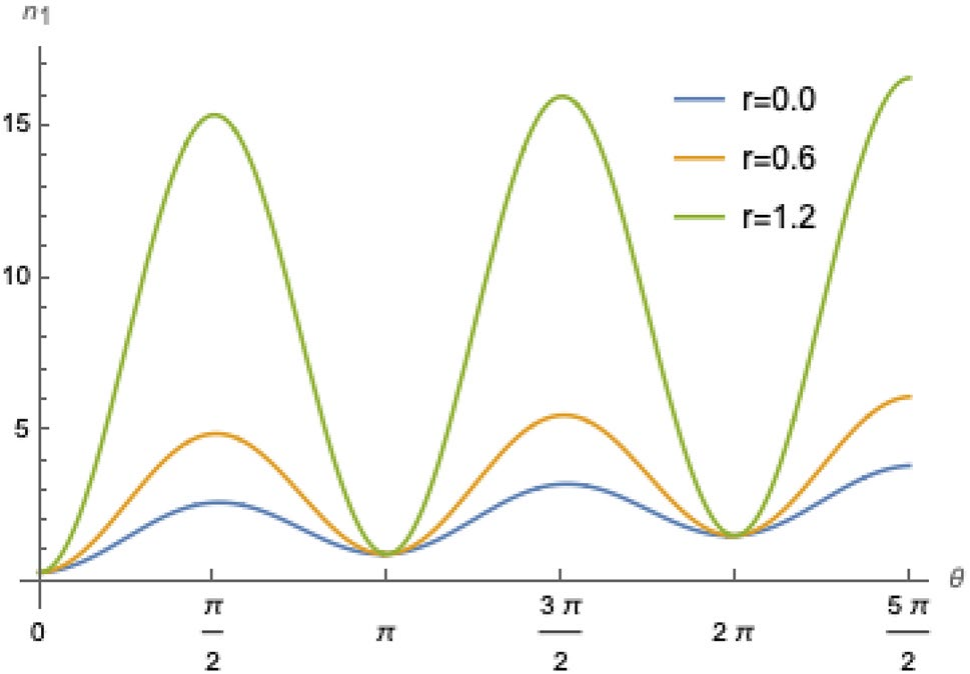
Population of mode 1, as function of $\theta =gt$ for ${\bar{n}}_1=0.35$ ($T_{1}=1.42\times 10^{-4}K$), ${\bar{n}}_2=2.3$ ($T_{2}=5.32\times 10^{-4}K$), $\Omega /2\pi = 4$ MHz, $g/\pi = 3.1$ kHz, and a heating rate $\Gamma =1.885$ quanta per milisecond, such that $\Gamma /g=0.1936$^37,57^.

Figure 7 shows the population of mode 1, Eq. (57), as function of time and some values of squeezing parameter *r* including a heating rate of 1.885 quanta per milisecond estimated in^37^ and is in perfect accordance with the experimental and simulated results obtained in Fig. 3 of that reference for $r=0$. We see an exchange of excitations between the two motional populations with a steady linear increase due to the heating. For $r\ne 0$, there is a pronounced rise in the population, particularly at $\pi /2$, when the two states are swapped, which is due to the squeezing in mode 2. The increase in population is more evident at $r=1.2$, as the initial population of mode 2 increases with $\cosh (2r)$.

In this situation, the Fisher information must be calculated following the same procedures of the previous section, but using Eq. (57) instead. For $r=0$, now the Fisher information elements of Eqs. (33) read

58 $$\begin{aligned} F_{\alpha \beta } =\frac{(\hslash \Omega /k_{B})^2}{T_{\alpha }^2 T_{\beta }^2} \frac{{\overline{n}}_{\alpha }{\overline{n}}_{\beta }(1+{\overline{n}}_{\alpha })(1+{\overline{n}}_{\beta }) \cos ^{2}{(gt+\frac{\pi }{2}\delta _{\alpha 2})} \sin ^2{(gt+\frac{\pi }{2}\delta _{1 \beta })} }{\left[ {\overline{n}}_{1}\cos ^{2}{(gt)}+ {\overline{n}}_{2}\sin ^2{(gt)}+ \Gamma t\right] \left[ 1+{\overline{n}}_{1}\cos ^2{(gt)}+ {\overline{n}}_{2}\sin ^2{(gt)}+\Gamma t\right] },\,\,\,\alpha , \beta = 1,2. \end{aligned}$$

**Figure 8:**
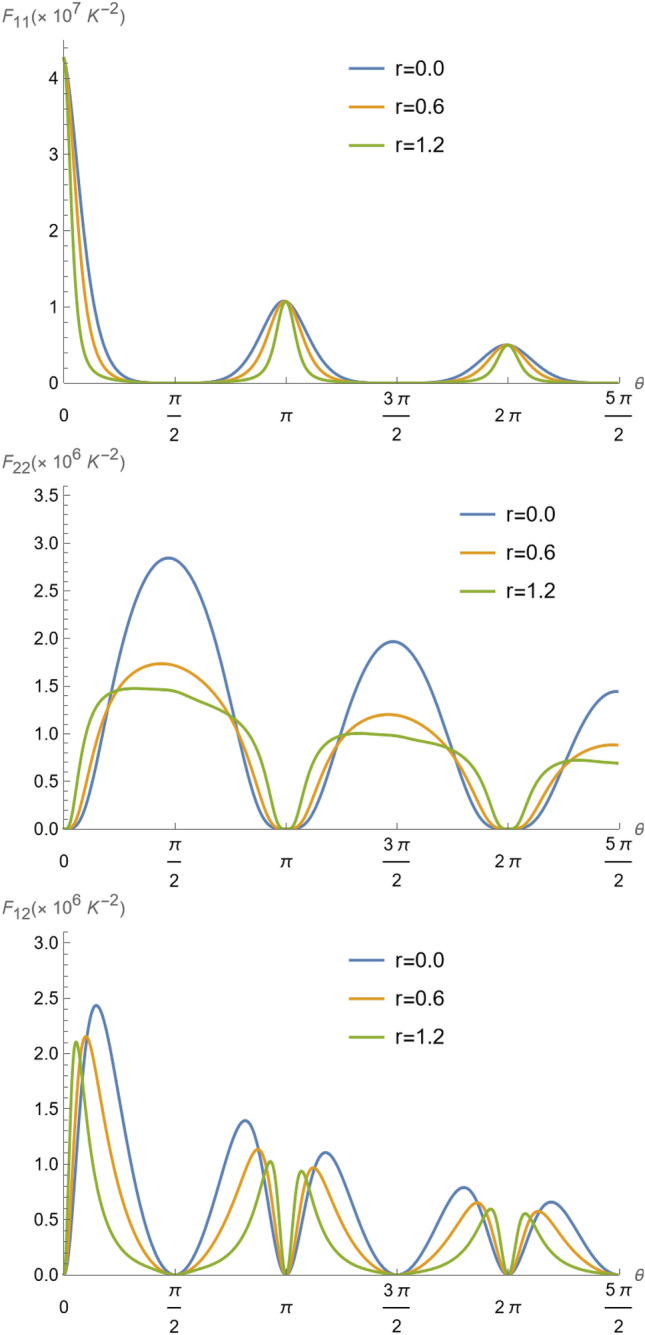
Fisher information $F_{11}$, $F_{22}$ and $F_{12}$ as a function of parameter $\theta =gt$ for ${\bar{n}}_1=0.35$ ($T_{1}=1.42\times 10^{-4}K$), ${\bar{n}}_2=2.3$ ($T_{2}=5.32\times 10^{-4}K$), $\Omega /2\pi = 4$ MHz, $g/\pi = 3.1$ kHz, and a heating rate $\Gamma =1.885$ quanta per milisecond ( $\Gamma /g=0.1936$ )^37,57^.

In Fig. 8, similarly to Fig. 4, we plot the Fisher information $F_{11}$, $F_{22}$ and $F_{12}$ evolution with $\theta =gt$, for the thermal and non-thermal reservoirs. We can see that the heating reduces the Fisher information with time, meaning that it introduces noise (dispersion) in the temperatures inference, as the individual temperatures increase. However, the noise effect is not substantial to hinder the inference of the ions temperatures at the heating rate of 1.885 quanta per milisecond. In fact, we observed that even for a heating rate of ten times the experimentally observed rates, the procedure is still useful to infer the temperature of mode 2 by measuring the excitations of mode 1. The same conclusions related to the spreading of the distributions and the advantage of the inference procedure without noise in “Fisher information” section, remain valid with noise. The only pronounced effect is to diminish the previously available periodicity for inference. Since the heating effect is more pronounced as the time goes on, the amplitude of the Fisher information peaks decrease with time, as $(\Gamma t)^{-2}$ from Eq. (58), which means that the heating effect introduces a dispersion, increasing with time in the inference of the temperatures. Also, we should remark, that the profiles of the Fisher information as the temperature of ion 2 is changed (Fig. 5) is not significantly changed, as it evaluates the best regimes for the inferred temperatures. This further confirms what we explained above.

**Figure 9:**
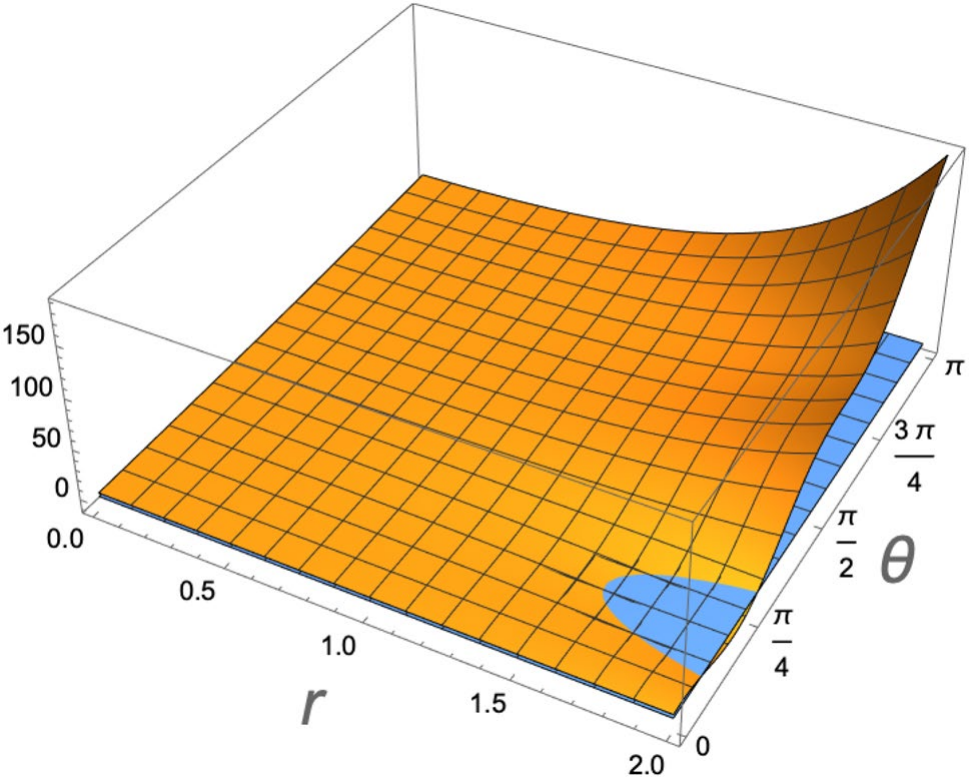
Entanglement of the ions logitudinal vibrational as a function of the squeezing parameter of ion 2, *r*, and the interaction $\theta =gt$, at a heating rate $\Gamma =1.885$ quanta per milisecond ($\Gamma /g=0.1936$). The entanglement is severely affected by the heating as the global state becomes more classical.

The only remaining question is how the entanglement between the vibrational modes of the two ions is affected by the heating effect. In Fig. 9 we see that it is severely affected by the heating effect. This occurs, despite the fact that only the populations of the modes, and not the correlations, are affected by the linear heating at the investigated timescale. The heating increases the modes populations linearly with time, while the other correlations are not affected. Therefore, the global system state becomes more classical. For the state to become entangled, it would be necessary to have higher values of *r* at disposition initially. Since the temperature estimation here presented, is not strictly dependent on the entanglement between the vibrational modes (available due to the squeezing), it is clear why, the Fisher information in Fig. 8 is not so severely affected. As a last remark we should mention that if other additional decoherence mechanism were present at the considered timescale, such as the dephasing we discussed before, the coherences would decay, and the global state would become even more classical, as if the squeezing parameter were smaller. This however only affects the entanglement and do not affect the strategy at the relevant timescale.

## Concluding remarks

In summary, we have explored the interaction between trapped ions for simultaneous inference of their vibrational temperatures. For entanglement analysis and temperature estimation, we take ion 1 with a thermal initial state, and ion 2 with the initial state to be thermal and non-thermal (due to quadrature squeezing). We use the interaction to effectively infer the temperature of the individual ions, by optimizing the interaction time evolution. It was observed that while the estimation of the temperature of ion 2 is slightly improved when the initial state is non-classical, the entanglement is not an essential ingredient, since what really matters is the ions state exchange, resulting from their interaction. Simultaneous measurements are often difficult to implement, in particular when associated with motional degrees of ions. The most practical way is to estimate the temperature by measuring each ion. Here, we chose to measure the ion that does not contain a possible squeezing, making it as a classical channel probe, contrary to what it has in the literature, where they use detectors with quantum properties for estimating parameters of classical systems^7,63–65^. To appropriately consider actual experimental conditions we have included a heating effect which is observed to occur due to surface-electrode noise. For that we developed a Langevin equation, considering two uncorrelated noise sources. The noise induces a linear (with time) heating effect, in accordance with experimental observations. Remarkably, although the noise, in the regime employed, decreases the entanglement of the joint vibrational degrees o freedom, it does not disturb considerable the Fisher information. This means that the method employed for temperature inference is useful, being robust against noise. Finally, we should remark that in trapped ion systems, thermal, displaced, squeezed and other arbitrary states were already generated, and therefore can be identified^41,42^. Although we considered only thermal, and squeezed thermal reservoirs, the same procedure would work for any arbitrary state. Moreover, the present strategy can be extended to remotely access other statistical properties of the ions.
